# WEALTH DISPARITIES IN LATER LIFE: SENSE OF MEANING AND PURPOSE IN LIFE

**DOI:** 10.1093/geroni/igae098.0970

**Published:** 2024-12-31

**Authors:** Shinae Choi

**Affiliations:** University of Alabama, Tuscaloosa, Alabama, United States

## Abstract

This study examined the extent to which racial/ethnic, gender, and age disparities in wealth persist among U.S. older adults, focusing on housing wealth (non-liquid assets) and non-housing wealth (liquid assets), alongside income, and further evaluated whether sense of meaning and purpose in life mitigates these wealth disparities in later life. Data were from the Leave-Behind Questionnaire in the 2018 Health and Retirement Study (N=4,472). I estimated ordinary least squares regression models with interaction terms to evaluate moderation effects, adjusting for health and sociodemographic characteristics. Results showed that wealth disparities across race/ethnicity, gender, and age groups exist in older Americans. Non-Hispanic Black, non-Hispanic Asian/other races, and Hispanic older adults compared to non-Hispanic Whites, as well as women (vs. men), reported significantly lower wealth and income. The age groups of 65-79 and 80+ reported higher wealth than those aged 50-64. Multiple moderation analyses revealed evidence of racial/ethnic and age disparities in wealth, but not gender, were mitigated by sense of meaning and purpose in life. Specifically, non-housing wealth increased among non-Hispanic Asian/other and Hispanic older adults as their purpose in life increased. A notable increase in both housing and non-housing wealth, as well as income, was observed among those aged 50-64 as their purpose in life increased. Findings suggest the protective effect of sense of meaning and purpose in life in mitigating wealth disparities in later life. Through further research and interventions aimed at bolstering sense of meaning and purpose in life, there is potential to alleviate wealth disparities in later life.

